# Associations of genetically predicted circulating levels of cytokines with telomere length: a Mendelian randomization study

**DOI:** 10.3389/fimmu.2023.1276257

**Published:** 2023-10-24

**Authors:** Renbing Pan, Mingjia Xiao, Zhigang Wu, Jingwen Liu, Lijun Wan

**Affiliations:** ^1^ Department of Urology, The Quzhou Affiliated Hospital of Wenzhou Medical University, Quzhou People’s Hospital, Quzhou, Zhejiang, China; ^2^ Department of Hepatology, The Quzhou Affiliated Hospital of Wenzhou Medical University, Quzhou People’s Hospital, Quzhou, Zhejiang, China; ^3^ Department of Urology, The First Affiliated Hospital of Wenzhou Medical University, Wenzhou, Zhejiang, China; ^4^ Department of Psychiatry, Shulan Quzhou Hospital, Quzhou, Zhejiang, China

**Keywords:** telomere length, cytokine, genome-wide association study, Mendelian randomization, single nucleotide polymorphisms

## Abstract

**Background:**

Telomere length (TL) has been regarded as a biomarker of aging, and TL shortening is associated with numerous chronic illnesses. The mounting evidence has shown that inflammatory cytokines are involved in maintaining or shortening TL, the causality of cytokines with TL remains unknown. Therefore, we performed a two-sample Mendelian randomization (MR) analysis to estimate the underlying correlations of circulating inflammatory cytokines with TL.

**Methods:**

Genetic instrumental variables for inflammatory cytokines were identified through a genome-wide association study (GWAS) involving 8,293 European individuals. Summary statistics of TL were derived from a UK Bio-bank cohort comprising 472,174 samples of individuals with European descent. We employed the inverse-variance weighted (IVW) approach as our main analysis, and to ensure the reliability of our findings, we also conducted additional analyses including the weighted median, MR-Egger, MR pleiotropy residual sum and outlier test, and weighted model. Lastly, the reverse MR analyses were performed to estimate the likelihood of inverse causality between TL and the cytokines identified in the forward MR analysis. Cochran’s Q test were employed to quantify the degree of heterogeneity.

**Results:**

After applying Bonferroni correction, a higher circulating level of Interleukin-7 (IL-7) was suggestively associated with TL maintaining (OR:1.01, 95%CI:1.00-1.02, P=0.032 by IVW method). The study also revealed suggestive evidence indicating the involvement of Interleukin-2 receptor, alpha subunit (IL-2Rα) level was negatively associated with TL maintaining (OR:0.98, 95%CI:0.96-1.00, P=0.045 by IVW method), and the weighted median approach was consistent (OR:0.99, 95%CI:0.97-1.00, P=0.035). According to the findings of reverse MR analysis, no significant causal relationship between TL and cytokines was explored. Our analysis did not reveal any substantial heterogeneity in the Single nucleotide polymorphisms or horizontal pleiotropy.

**Conclusions:**

Our MR analysis yielded suggestive evidence supporting the causality between circulating IL-7 and IL-2Rα and telomere length, necessitating further investigations to elucidate the mechanisms by which these inflammatory cytokines may impact the progression of telomeres.

## 1 Introduction

Telomeres are specific nucleoprotein structures composed of repetitive TTAGGG sequences at the end of linear chromosomes (1). Due to their critical biological character in maintaining chromosomal stability and integrity, preventing coalescence of chromosomal ends, and assessing cell proliferation (2). Telomeres have been widely recognized as a reliable biomarker for assessing survival, pressure, and senility (3–5). Telomere shortening is associated with an elevated risk of mellitus (6), malignant tumor (7), cardiovascular diseases (8), and obesity (9).

Previous studies have suggested that the acceleration of telomere shortening is primarily attributed to oxidative stress and inflammation, which facilitate cell renewal and promote the duplication of senescence cells (10–12). Moreover, the process of inflammation stimulates the generation of reactive oxygen species (ROS), resulting in telomere DNA damage, which subsequently leads to a loss of replication ability and accelerates cellular aging and apoptosis. Additionally, the consequence of this is the impairment of stem and progenitor cell function, resulting in functional recession and tissue atrophy (13, 14). Recently, accumulating evidence has shown that circulating inflammatory cytokines might be correlated with shorter telomeres. M. Groer et al. reported a negative association between telomere length shortening and IL-6 levels, which serves as a representative marker for inflammatory response (15). They reported that senescent cells not only shortened telomeres, but also caused overexpression of the transcription factor nuclear factor-kB during cell senescence, and these leaded to overproduction of circulating cytokines including IL-6, TNF-α, and macrophage interferon (16). Interestingly, an association has been also observed between hypoadiponectinemia and telomere shortening in obesity (17). What’s more, the verisimilitude of telomere DNA damage being related to the ascent of CRP levels has been supported by several epidemiological data (18). Another research conducted by T. Wang et al. revealed a natural correlation between telomere shortening-induced aging and the elevation of proinflammatory cytokines levels, which in turn stimulate the development of COPD through an inflammatory response (19). Considering the susceptibility of observational studies to potential control bias and reversed causality (20), further exploration into the underlying causal correlation between circulating levels of cytokines and telomere was still required.

Mendelian randomization (MR) study is a robust approach that utilizes single nucleotide polymorphisms as instrumental variables (IVs) to assess the causal link between exposures and outcomes (21). The evidence of causality provided by the MR research was reported to lie at the interface between conventional epidemiological studies and randomized controlled trials (RCTs) (22). Recently, a meta-analysis of genome-wide association study (GWAS) was conducted to assess the genetic basis for 41 circulating cytokines, providing an opportunity to explore their potential association with telomere length. Hence, by performing a two-sample MR analysis, we systematically evaluated the underlying causal associations between circulating levels of cytokines and telomeres.

## 2 Materials and methods

### 2.1 Study design

**Figure 1:**
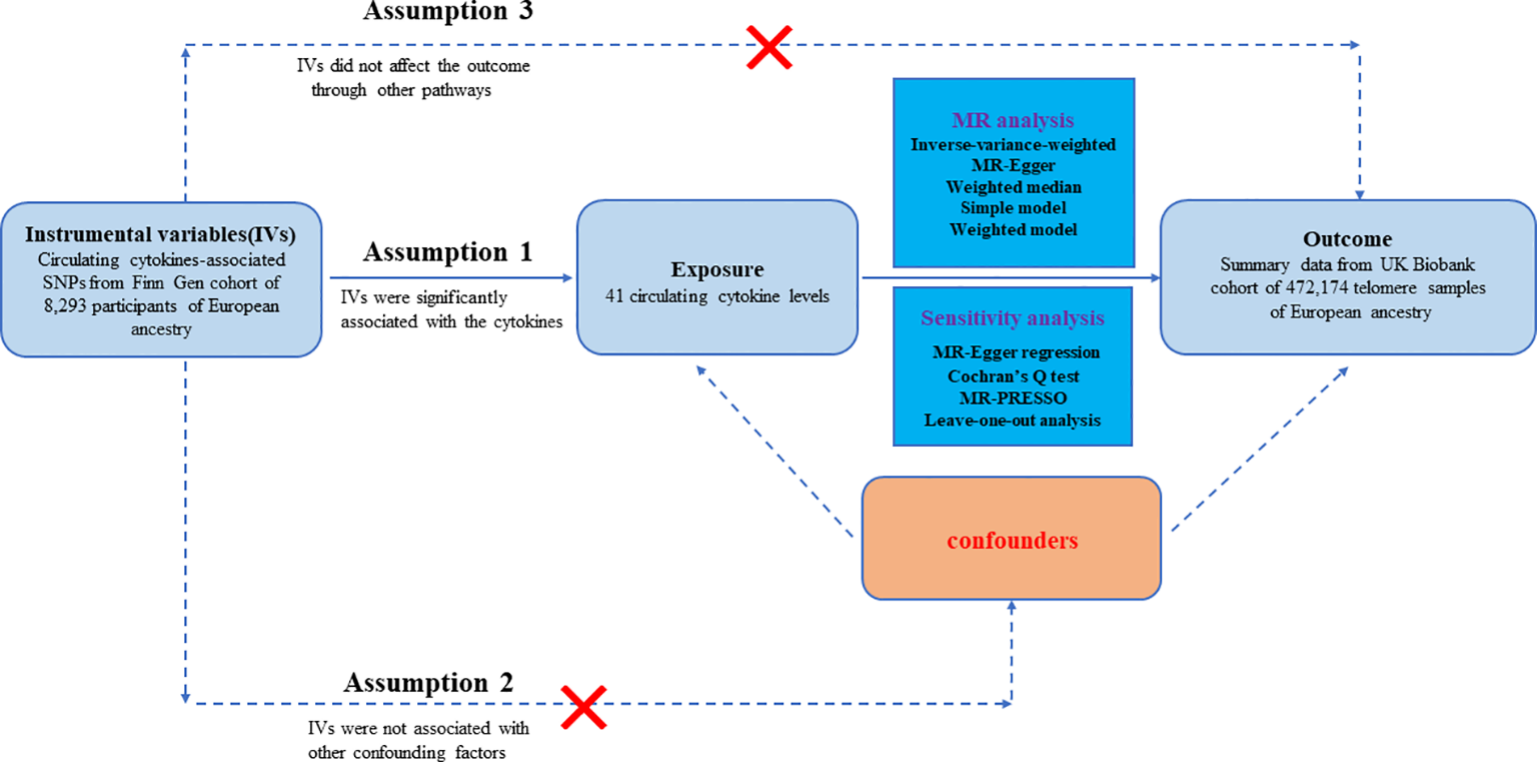
Datasets, assumptions, and study design of two-sample Mendelian randomization for circulating levels of 41 cytokines and telomere length.

The workflow of our study is displayed in **Figure 1**. The study was on a basis of publicly available data from GWAS on inflammatory cytokines and telomere length, with explicit characteristics listed in **Supplementary Table S1**. To enhance the reliability of the MR analysis findings, this study endeavored to fulfill the following three assumptions. Firstly, the IVs are strongly associated with circulating levels of cytokines. Secondly, no confounders are related to the IVs. Thirdly, the IVs influence the outcome only via exposure and there are no other causal pathways for the IVs to influence the outcome (23). We extracted genetic IVs for each circulating cytokine to explore the causal link from each inflammatory cytokine to telomeres. The utilization of publicly available GWAS summary datasets obviated the need for ethical approval.

### 2.2 Data sources and instruments

#### 2.2.1 Cytokines

We collected genome-wide association summary datasets for 41 circulating inflammatory cytokines from the most proximate GWAS reported by Ahola-olli et al. (24), including 8,293 Finns from three independent cohorts: FINRISK1997, FINRISK2002, and the Young Finns Cardiovascular Risk Study (YFS). These 41 circulating cytokine distributions were normalized by converse transformation. An additive genetic model, adjusted for sex, age, body mass index (BMI), and the first ten genetic principal components, was employed to examine univariable correlations between 10.7 million genetic polymorphisms and 41 circulating cytokine concentrations.

Firstly, the genome-wide significant threshold of p<5e-08 was applied to each of the 41 circulating cytokines in order to identify robust IVs associated with their levels. Due to no or few (<3) IVs were extracted for a majority of the cytokines at the p-value<5e-08 level, we widen the threshold to p-value<5e-06 to select eligible IVs. Lastly, all 41 circulating cytokines were identified under this standard. Secondly, the impact of robust linkage disequilibrium (LD) between SNPs was mitigated by utilizing a LD threshold for the selected SNPs(r²<0.001,10000kb), ensuring independence among instrumental variables for each exposure. Thirdly, to avoid weak instrument bias, the average of SNPs F-statistics was computed (25, 26), and the F-statistics greater than 10 were regarded as robust IVs for our study. The F-statistic is a statistic approach that captures the magnitude and accuracy of the genetic effect on the trait. It can be calculated as F=R^2^(N-2)/(1-R^2^), where R^2^ represents the proportion of variance in the trait illuminated by the SNP, and N denotes the sample size of GWAS involving SNPs with the trait (27). Fourthly, intermediate allele frequency palindromic SNPs were excluded because allele frequencies were not provided in the GWAS of circulating cytokines, so we could not determine whether these SNPs were consistent with the direction of exposure and outcome. Additionally, to satisfy the independence assumption of MR, SNPs associated with confounders, including blood pressure, body mass index (BMI), and smoking, were excluded by applying the PhenoScannerV2 online platform. Furthermore, we performed a phenome-wide association study (including alcohol consumption, smoking, exercise, instant coffee intake, physical activity, sedentary, hypertension, atrial fibrillation, diabetes mellitus, and serum urate level, which can affect telomere length) of the instrumental variables. Finally, altogether 450 SNPs related to 41 cytokines were extracted as IVs in our study. The figure and F-statistics of the SNPs which are utilized in this study are displayed in **Supplementary Table S2**.

#### 2.2.2 Telomere length

The genetically-related datasets for telomeres were selected from the publicly attainable GWAS (28), which was implemented using 488400 DNA samples from UK Biobank (UKB) participants. Mean telomere length was calculated utilizing an unequivocal quantitative PCR assay and underwent comprehensive quality inspection and technical change adjustments. Ultimately, 472,174 telomere length measurements were retained for our MR analysis. The individuals in the UK Biobank study were exclusively participants aged between 40 and 69 years, with a parallel percentage of men (45.8%) and women (54.2%). For reverse MR analyses, in all 117 genome-wide significant (p<5e-08) SNPs were authenticated for TL. Then, these IVs were fetched to assess the impact of genetic predisposition to TL on circulating cytokines.

### 2.3 Statistical analyses

To detect the causal effects of circulating levels of cytokines on TL by conjoining diverse SNPs, we performed a two-sample Mendelian randomization analysis employing five commonly employed analytical means, including MR-Egger, Weighted median, Inverse variance weighted (IVW), Simple mode, and Weighted mode (29, 30). The random-effects IVW approach is a dominant statistic method for aggregating Wald ratio estimations of various SNPs, exhibiting the highest statistic power among various MR methods. This method was recognized as the primary approach evaluating the underlying causal links between circulating cytokines levels and TL. Our analysis necessitated data on SNPs, alleles, effect sizes, P-values, and allele frequencies (EAF) (31). The weighted median estimator can yield reliable estimates even when incorporating 50% of the noneffective genetic instruments. Due to its widened threshold, the MR-Egger method yields valid estimates even in the presence of horizontal pleiotropy among SNPs, where all instrumental variables are invalid. Additionally, simple mode and weighted mode were also applied as auxiliary analysis methods.

### 2.4 Sensitivity analyses

The sensitivity analyses were executed by MR pleiotropy residual sum and outlier (MR-PRESSO), MR-Egger regression, Cochran’s Q Test, and leave-one-out analysis. Firstly, we utilized the simple-median method and weighted-median method to evaluate the underlying causal effects in situations where conventional assumptions were challenged (32). Secondly, The MR-Egger regression was conducted to assess the presence of horizontal pleiotropy, with statistical significance defined as p-values for the intercept being less than 0.05 (33). Thirdly, the MR-PRESSO global test was applied to investigate potential outliers as plausible pleiotropic biases and mitigate the impact of pleiotropy by excluding the specific SNPs that deviated from normality. Finally, the Cochran’s Q statistic was utilized for IVW and MR-Egger to estimate heterogeneity among SNPs. A p-value greater than 0.05 in the Cochran’s Q test indicated the absence of heterogeneity among the IVs (32).In addition, we further examined whether some SNPs could affect the results independently and evaluated the stability of effect sizes via leave-one-out analysis. Furthermore, the Bonferroni correction was utilized to resolve the issue of multiple comparisons, and a significance level of p<0.00024 (0.05/(41*5)) was adopted (Bonferroni correction with 41 tests* 5 models).

All the MR analysis and sensitivity analysis were performed with R (version 4.2.3). R packages “Two Sample MR” and “MR-PRESSO” packages were utilized.

## 3 Results

**Figure 2:**
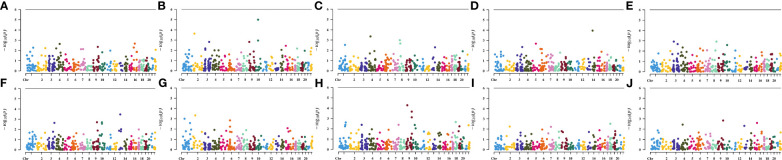
Manhattan plot of genetically predicted circulating levels of cytokines on relevant phenotypes affecting telomere length, **(A)** alcohol consumption, **(B)** smoking, **(C)** exercise, **(D)** instant coffee intake, **(E)** physical activity, **(F)** sedentary, **(G)** hypertension, **(H)** atrial fibrillation, **(I)** diabetes mellitus, and **(J)** serum urate.

Firstly, the MR analysis was conducted by expanding the threshold to p-value<5e-06, taking account of the limited genetic variance, as well as the restricted number of SNPs and low statistical powers. By utilizing this standard (r²<0.001, p<5e-06), PhenoScannerV2 database screening, and removing the intermediate allele frequency palindromic SNPs, altogether 450 SNPs associated with 41 circulating cytokines were extracted. The F-statistics of each SNP employed in this study ranged from 20.01 to 345, indicating the robustness and strength of the IVs. In addition, we conducted a phenome-wide association study of the instrumental variables. We found no SNPs showing an association p-value<5e-06 with those relevant phenotypes affecting telomere length (including alcohol consumption, smoking, exercise, instant coffee intake, physical activity, sedentary, hypertension, atrial fibrillation, diabetes mellitus, and serum urate level), indicating that no potential confounding effect on results. The results of phenome-wide association study were shown in **Figure 2**.

**Figure 3:**
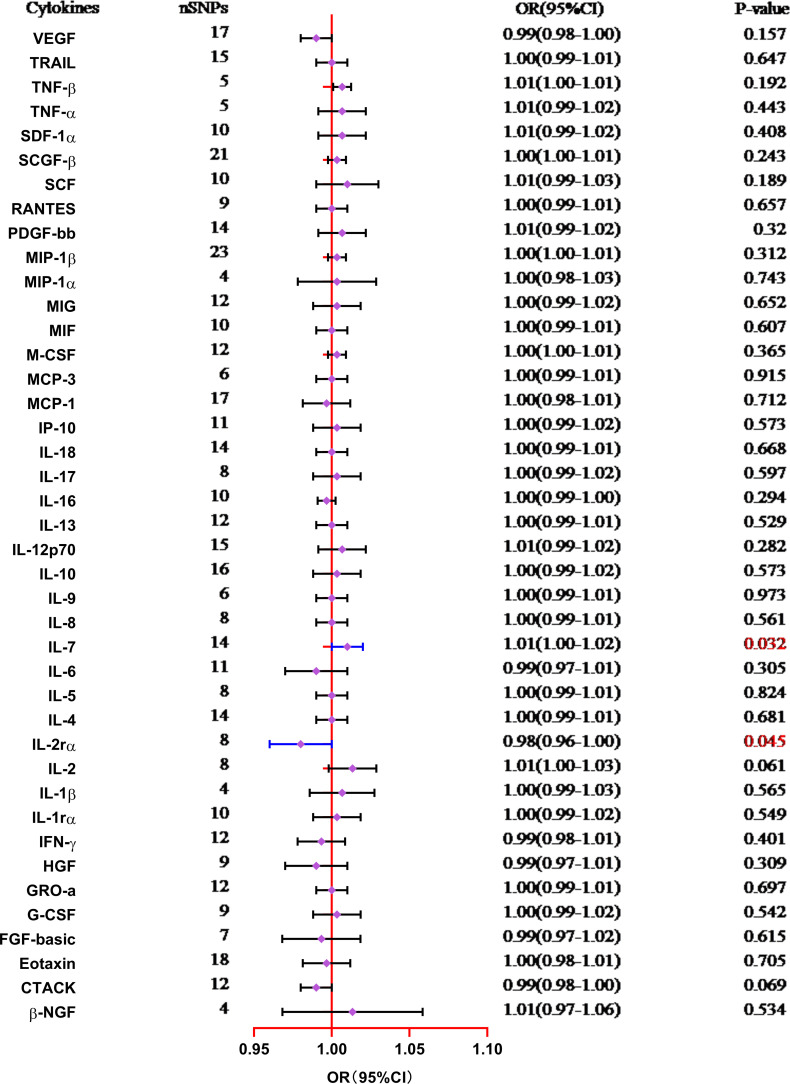
Forest plot of the Mendelian randomization analyses for the associations between circulating levels of cytokines and telomere length (IVW method). CI, confidence interval; OR, odds ratio; SNP, single nucleotide polymorphism.

**Figure 4:**
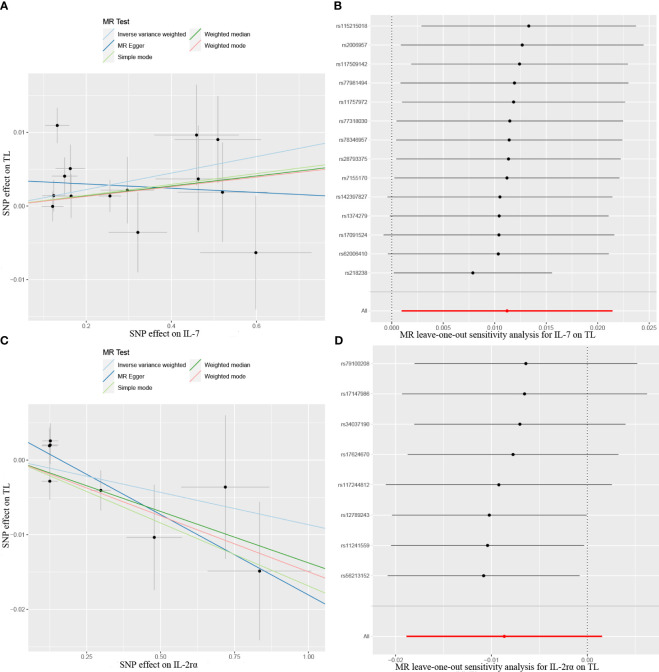
Scatter plots of Mendelian randomization (MR) analyses between circulating levels of cytokines and telomere length (TL), **(A)** IL-7; **(C)** IL-2rα. MR leave-one-out sensitivity analysis to assess whether every single SNP drove the causal association of cytokines on TL, **(B)** IL-7; **(D)** IL-2rα. SNP, single nucleotide polymorphism.

The results from the MR analysis of the associations between 41 cytokines and TL are shown in **Figure 3** and **Supplementary Table S3**. After applying the Bonferroni correction, only two cytokines (IL-7 and IL-2Rα) manifested suggestive associations with TL. The explicit characteristics for the associated SNPs were summarized in **Supplementary Table S4**. Genetically predicted elevation in circulating IL-7 levels exhibited a suggestively positive association with TL (OR:1.01, 95%CI:1.00-1.02, P=0.032 by IVW method). The findings of the alternative approach demonstrated comparable trends, although they did not reach statistical significance (OR:1.01, 95%CI:1.00-1.02, P=0.227 by Weighted median method; OR:1.00, 95%CI:0.98-1.02, P=0.783 by MR Egger method). Scatter plots of associations of genetically predicted IL-7 levels with TL were presented in **Figure 4A**. The leave-one-out approach was employed for sensitivity analysis and exhibited no impact in **Figure 4B**. The MR-Egger regression analysis did not investigate underlying directional pleiotropic effects among the SNPs (intercept p-value=0.139).

The findings of our study additionally manifested a suggestive inverse association between the circulating levels of IL-2Rα and TL (OR:0.98, 95%CI:0.96-1.00, P=0.045 by IVW method), and the weighted median approach was in accord with IVW method (OR:0.99, 95%CI:0.97-1.00, P=0.035), but the MR-Egger approach was non-significant (OR:0.99, 95%CI:0.98-1.00, P=0.096). Scatter plots for five methods highlighted the effect of IL-2Rα on TL in **Figure 4C**. Furthermore, the absence of pleiotropy was supported by the non-significant intercept observed in MR-Egger regression analysis (intercept p-value=0.113). The results of the leave-one-out method similarly indicated that none of the individual SNPs exerted significant influence on the analysis outcomes (**Figure 4D**).

The absence of evidence supported the lack of causal associations between other cytokines and TL (**Supplementary Table S3**). The MR-PRESSO global test showed no pleiotropy between the IVs and outcomes (**Supplementary Table S5**). Furthermore, MR-Egger and IVW Cochran’s Q tests indicated no significant heterogeneity of IVs (all p-values>0.05). The reverse MR analysis utilizing the IVW method did not identify any statistically significant associations of TL on IL-7 (OR:0.95, 95%CI:0.78-1.16, P=0.647) and IL-2Rα (OR:0.85, 95%CI:0.70-1.03, P=0.104) levels. The IVs we utilized and the outcomes of alternative approaches are documented in **Supplementary Table S6**.

## 4 Discussion

In this study, we conducted a two-sample Mendelian randomization approach to explore the underlying causal associations between circulating levels of 41 cytokines and TL. We have discovered suggestive evidence indicating a causal association between genetically predicted levels of circulating IL-7 and IL-2rα and TL. Nevertheless, we did not find any evidence indicating a causal relationship between the levels of other circulating cytokines and TL.

The cytokine IL-7 belongs to the cytokine family and possesses four anti-parallel α helixes that interact with Type I cytokine receptors. The production of this substance which plays a pivotal role in stimulating growth and maintaining homeostatic of lymphoid cells is attributed to stromal cells (34). Reports that have observed an association between IL-7 levels and maintaining telomere length are few. Previous experimental studies manifested that the cells stimulated by IL-7 or IL-15 maintain their naïve phenotype and preserve telomere length through the increased telomerase activity (35). These observational research results indicated that IL-7 was a potential protective factor for telomere length maintaining. Coincidentally, we observed a causal association between higher genetically predicted circulating IL-7 level and telomere maintenance, which aligns with previous study findings. The findings of our study were substantiated by the evidence obtained from experimental research through MR analysis. It has been reported that IL-17 exerts concentration, time, and subset-dependent effects by accelerating cell proliferation and protecting cells from apoptosis. Furthermore, long-drawn exposure of naïve CD8^+^ T cells to IL-7 facilitates proliferation without inducing differentiation or shortening of telomeres (35). These results suggested that IL-7 may serve as an underlying therapeutic target in maintaining telomere length, but further investigation to verify the potential biological mechanism were required.

The transmembrane protein IL-2Rα, also called CD25, is expressed on the surface of activated B cells, T cells, oligodendrocytes, myeloid precursors, and thymocytes (36). Additionally, human resting memory T cells constitutively expressed CD25 (36). The signaling pathways mediated by IL-2/IL-2Rα (CD25) are crucial in the regulation of adaptive immune reaction and the control of T cell proliferation and survival (37). Nevertheless, the epidemiological evidence for the association between IL-2Rα and telomere length was few, limited by employing a case-control study design and restricted by diminutive sample sizes. Previous research conducted by F. Albarran-Tamayo et al. (38) showed that the analysis did not reveal any significant correlation between IL-2Rα expression levels in circulation and telomere length shortening or reduced T cell proliferation. However, our results indicated that genetically predicted circulating IL-2Rα level was negatively associated with telomere length, indicating that the findings of observational studies may require validation through additional research.

In our study, the links between multiple circulating cytokines and telomere length were assessed utilizing MR analysis. Previously, S. Li et al. confirmed that the length of telomere exhibited a positive correlation with the expression levels of IL-6 and MIP-1α in bone marrow mesenchymal stem cells (MSCs) derived from patients with multiple myeloma (MM) (39). They found that the increase in telomerase activity induced by IL-6 was demonstrated to occur through AKT-mediated phosphorylation of hTERT in MM cell lines, without any variation observed in the expression of hTERT at the mRNA or protein level (40), suggesting that the biological mechanism employed by MSCs to maintain their telomere length in an inflammatory cytokine-rich microenvironment, such as IL-6, may also be utilized (39). In addition, they also reported that the contribution of MIP-1α to the progression of bone disease in MM is manifested through its promotion of tumor survival (41), inhibition of osteoblast function (42), and modulation of osteoblast differentiation (43). Moreover, the expression of MIP-1α in MM-MSCs was found to be significantly higher compared to MSCs from the control group, suggesting that MM-MSCs may play a vital role in the initiation and progression of MM (39). These results indicate that IL-6 and MIP-1α are protective factors for maintaining telomere length. However, in this study, we did not find any evidence indicating a correlation between higher genetically predicted levels of circulating IL-6 or MIP-1α and the maintenance of telomere length. Moreover, another study reported by R. M. Corbo et al. (44) showed that a significant negative linear correlation was observed between serum IL-1β levels and telomere length, after adjusting for age. Nevertheless, our study did not find a link between serum IL-1β levels and telomere length. The variations could be attributed to the diverse choice of IVs and GWAS summary data. Given the inconsistency of the results, more studies are needed to detect the accurate potential biological mechanisms of these cytokines in maintaining or shortening telomere length. For as we know, this is the first comprehensive and systematic MR analysis of the associations between circulating levels of cytokines and telomere length. Lastly, the reverse MR analysis we conducted yielded robust evidence to substantiate our primary research.

However, our study was subject to several limitations. First, the selection of IVs was conducted using a relaxed significance threshold of p<5e-06, and this might lead to consequence bias and false-positive variants. Nonetheless, the F-statistics of IVs all exceeded 10, indicating minimal presence of weak instrument bias. Similarly, several previous studies have also utilized the identical threshold (p<5e-06) when assessing the associations between inflammatory cytokines and systemic lupus erythematosus (45). Second, the participants of GWAS included in our study were limited to European descent, and it remains highly questionable whether the same results can be extrapolated to other races. Third, although pleiotropy cannot be completely ruled out, we have taken measures to exclude SNPs associated with underlying confounding factors and performed multiple sensitivity analyses using different assumptions, such as MR-Egger regression. Fourth, after applying the Bonferroni correction, no cytokines demonstrated a statistically significant correlation with telomere length, and the associations of only two cytokines (IL-7 and IL-2Rα) were found to be suggestive. Finally, MR analyses are not equal to a randomized controlled trial (RCT), the involvement of cytokines in maintaining or shortening of telomere length may not be causally established. Thus, these potential associations need to be validated in larger cohorts and the underlying involvement of cytokines in the regulation of telomere length maintenance or shortening should be further explored in future studies.

## 5 Conclusions

Our study provided support for the underlying causal associations between two circulating cytokines (IL-7 and IL-2Rα) and telomere length. Further investigations are warranted to verify these findings, elucidate potential biological mechanisms, and evaluate their utility as biomarkers and underlying therapeutic targets for telomere length development.

## Data availability statement

The original contributions presented in the study are included in the article/**Supplementary Material**. Further inquiries can be directed to the corresponding authors.

## Author contributions

RP: Conceptualization, Methodology, Writing – original draft. MX: Software, Writing – original draft, Funding acquisition. ZW: Visualization, Writing – review & editing. JL: Supervision, Writing – original draft. LW: Funding acquisition, Writing – review & editing.

## Glossary

**Table:**

β-NGF	beta nerve growth factor
CTACK	cutaneous T-cell attracting (CCL27)
FGF-basic	basic fibroblast growth factor
G-CSF	granulocyte colony- stimulating factor
GRO-a	growth regulated oncogene-α
HGF	hepatocyte growth factor
IFN-γ	interferon-gamma
IL-1rα	interleukin-1 receptor antagonist
IL-1β	interleukin-1-beta
IL-2	interleukin-2
IL-2rα	interleukin-2 receptor, alpha subunit
IL-4	interleukin-4
IL-5	interleukin-5
IL-6	interleukin-6
IL-7	interleukin-7
IL-8	interleukin-8 (CXCL8)
IL-9	interleukin-9
IL-10	interleukin-10
IL-12p70	interleukin-12p70
IL-13	interleukin-13
IL-16	interleukin-16
IL-17	interleukin-17
IL-18	interleukin-18
IP-10	interferon gamma-induced protein 10 (CXCL10)
MCP-1	monocyte chemotactic protein-1 (CCL2)
MCP-3	monocyte specific chemokine 3 (CCL7)
M-CSF	macrophage colony-stimulating factor
MIF	macrophage migration inhibitory factor
MIG	monokine induced by interferon-gamma (CXCL9)
MIP-1α	macrophage inflammatory protein-1α
MIP-1β	macrophage inflammatory protein-1β
PDGF-bb	platelet derived growth factor BB
RANTES	regulated on activation normal T Cell expressed and secreted (CCL5)
SCF	stem cell factor
SCGF-β	stem cell growth factor beta
SDF-1α	stromal cell-derived factor-1 alpha (CXCL12)
Eotaxin	(CCL11)
TNF-α	tumor necrosis factor-alpha
TNF-β	tumor necrosis factor-beta
TRAIL	TNF-related apoptosis inducing ligand
VEGF	vascular endothelial growth factor
