# Supplementary material for: Associations of genetically predicted circulating levels of cytokines with telomere length: a Mendelian randomization study
Source: Front Immunol. 2023 Oct 24;14:1276257. doi: 10.3389/fimmu.2023.1276257 (PMC10628532; doi:10.3389/fimmu.2023.1276257)
Supplement: Supplementary file 1 [file DataSheet_1.docx]

Supplementary Materials for

Associations of genetically predicted circulating levels of cytokines with telomere length: a Mendelian randomization study

Renbing Pan, Mingjia Xiao, Zhigang Wu, Jingwen Liu, Lijun Wan

**Table S1.** Brief characteristics description of 41 cytokines and telomere length GWAS cohorts involved in this study.

**Table S2.** Details of the number of genetic instruments and *F*-statistic for each cytokine and growth factor.

Abbreviations: No., number; SNP, single nucleotide polymorphism.

**Table S3.** Effect estimates of the associations between circulating levels of 41 cytokines and telomere length in MR analyses.

Abbreviations: β-NGF, beta nerve growth factor; CTACK, cutaneous T-cell attracting (CCL27); FGF-basic, basic fibroblast growth factor; G-CSF, granulocyte colony-stimulating factor; GRO-a, growth regulated oncogene-α (CXCL1); HGF, hepatocyte growth factor; IFN-γ, interferon-gamma; IL-1rα, interleukin-1 receptor antagonist; IL-1β, interleukin-1-beta; IL-2, interleukin-2; IL-2rα, interleukin-2 receptor, alpha subunit; IL-4, interleukin-4; IL-5, interleukin-5; IL-6, interleukin-6; IL-7, interleukin-7; IL-8, interleukin-8; IL-9, interleukin-9; IL-10, interleukin-10; IL-12p70, interleukin-12p70; IL-13, interleukin-13; IL-16, interleukin-16; IL-17, interleukin-17; IL-18, interleukin-18; IP-10, interferon gamma-induced protein 10 (CXCL10); MCP-1, monocyte chemotactic protein-1; MCP-3, monocyte specific chemokine 3 (CCL7); M-CSF, macrophage colony-stimulating factor; MIF, macrophage migration inhibitory factor; MIG, monokine induced by interferon-gamma; MIP-1α, macrophage inflammatory protein-1α (CCL3); MIP-1b, macrophage inflammatory protein-1β; MR, Mendelian randomization; MR-PRESSO,MR pleiotropy residual sum and outlier; OR, odds ratio; PDGF-bb, platelet derived growth factor BB; RANTES, regulated on activation normal T Cell expressed and secreted (CCL5); SCF, stem cell factor; SCGF-β, stem cell growth factor beta; SDF-1α, stromal cell-derived factor-1 alpha; SNP, single nucleotide polymorphism; TNF-α, tumor necrosis factor-alpha; TNF-β, tumor necrosis factor-beta; TRAIL, TNF-related apoptosis inducing ligand; VEGF, vascular endothelial growth factor; CI, confidence interval; OR, odds ratio; SNP, single nucleotide polymorphism. **P*-value of the intercept from MR-Egger regression analysis.

**Table S4.** Characteristics of instrumental variables used for circulating levels of IL-7 and IL-2Rα in this study.

Abbreviations: Chr, chromosome; IL-7, interleukin-7; IL-2Rα, Interleukin-2 receptor, alpha subunit; SE, standard error; SNP, single nucleotide polymorphism.

**Table S5.** The global test and causal estimate results of MR pleiotropy residual sum and outlier (MR-PRESSO).

**Table S6.** Effect estimates of the associations of telomere length with circulating levels of IL-7 and IL-2Rα.

Abbreviations: CI, confidence interval; IL-7, interleukin-7; IL-2Rα, Interleukin-2 receptor, alpha subunit; OR, odds ratio; SNP, single nucleotide polymorphism. **P*-value of the intercept from MR-Egger regression analysis.

**Table S1.** Brief characteristics description of 41 cytokines and telomere length GWAS cohorts involved in this study.

| Exposure or outcome | Source | Sample size | Ancestry | Access Link | PMID |
| --- | --- | --- | --- | --- | --- |
| Circulating levels of 41 cytokines and growth factors | FinnGen | 8,293 participants | European | http://computationalmedicine.fi/data#  Cytokine_GWAS | 27989323 |
| Telomere length | UK Biobank | 472,174 samples | European | http://gwas.mrcieu.ac.uk/datasets/ieu-b-4879/ | / |

**Table S2.** Details of the number of genetic instruments and *F*-statistic for each cytokine and growth factor. ***(P* < 5 ×10^-6^)**

| Cytokines/ Growth factors | Abbreviations | No. of SNPs | *F*-statistic (range) | Number |
| --- | --- | --- | --- | --- |
| Cutaneous T-cell attracting (CCL27) | CTACK | 12 | 22.92(20.84-31.44) | GCST004420 |
| Beta nerve growth factor | β-NGF | 4 | 24.59(20.83-35.43) | GCST004421 |
| Vascular endothelial growth factor | VEGF | 17 | 28.89 (20.88-71.72) | GCST004422 |
| Macrophage migration inhibitory factor (glycosylation-inhibiting factor) | MIF | 10 | 23.15(21.07-27.07) | GCST004423 |
| TNF-related apoptosis inducing ligand | TRAIL | 15 | 54.19 (20.83-345.00) | GCST004424 |
| Tumor necrosis factor-beta | TNF-β | 5 | 25.31 (20.98-38.27) | GCST004425 |
| Tumor necrosis factor-alpha | TNF-α | 5 | 22.46 (21.17-23.54) | GCST004426 |
| Stromal cell-derived factor-1 alpha (CXCL12) | SDF-1α | 10 | 22.46 (20.84-27.84) | GCST004427 |
| Stem cell growth factor beta | SCGF-β | 21 | 24.15 (20.83-42.10) | GCST004428 |
| Stem cell factor | SCF | 10 | 22.99 (21.13-25.82) | GCST004429 |
| Interleukin-16 | IL-16 | 10 | 28.99(21.21-53.45) | GCST004430 |
| Regulated on activation, normal T Cell expressed and secreted (CCL5) | RANTES | 9 | 22.97(20.84-27.21) | GCST004431 |
| Platelet derived growth factor BB | PDGF-bb | 14 | 24.24(21.14-31.12) | GCST004432 |
| Macrophage inflammatory protein-1β (CCL4) | MIP-1β | 23 | 28.34(20.95-87.99) | GCST004433 |
| Macrophage inflammatory protein-1α (CCL3) | MIP-1α | 4 | 21.96(20.94-24.11) | GCST004434 |
| Monokine induced by interferon-gamma (CXCL9) | MIG | 12 | 23.64(21.04-30.58) | GCST004435 |
| Macrophage colony-stimulating factor | MCSF | 12 | 21.96(20.92-25.52) | GCST004436 |
| Monocyte specific chemokine 3 (CCL7) | MCP-3 | 6 | 22.21(20.77-26.39) | GCST004437 |
| Monocyte chemotactic protein-1 (CCL2) | MCP-1 | 17 | 25.27(20.01-41.97) | GCST004438 |
| Interleukin-12p70 | IL-12p70 | 15 | 24.63(20.89-48.69) | GCST004439 |
| Interferon gamma-induced protein 10 (CXCL10) | IP-10 | 11 | 22.29(20.87-24.72) | GCST004440 |
| Interleukin-18 | IL-18 | 14 | 23.41(21.01-27.31) | GCST004441 |
| Interleukin-17 | IL-17 | 8 | 23.85(20.90-32.13) | GCST004442 |
| Interleukin-13 | IL-13 | 12 | 22.88(20.84-27.21) | GCST004443 |
| Interleukin-10 | IL-10 | 16 | 30.76(20.98-132.61) | GCST004444 |
| Interleukin-8 (CXCL8) | IL-8 | 8 | 21.95(20.89-23.85) | GCST004445 |
| Interleukin-6 | IL-6 | 11 | 22.77(20.77-30.71) | GCST004446 |
| Interleukin-1 receptor antagonist | IL-1rα | 10 | 21.94(20.77-24.25) | GCST004447 |
| Interleukin-1-beta | IL-1β | 4 | 23.00(21.79-25.53) | GCST004448 |
| Hepatocyte growth factor | HGF | 9 | 25.80(21.00-55.42) | GCST004449 |
| Interleukin-9 | IL-9 | 6 | 22.40(21.54-24.60) | GCST004450 |
| Interleukin-7 | IL-7 | 14 | 27.42(20.84-95.19) | GCST004451 |
| Interleukin-5 | IL-5 | 8 | 22.40(20.85-25.57) | GCST004452 |
| Interleukin-4 | IL-4 | 14 | 22.97(20.88-26.61) | GCST004453 |
| Interleukin-2 receptor, alpha subunit | IL-2Rα | 8 | 29.45(20.93-77.94) | GCST004454 |
| Interleukin-2 | IL-2 | 8 | 22.80(20.94-27.62) | GCST004455 |
| Interferon-gamma | IFN-γ | 12 | 23.16(21.47-26.14) | GCST004456 |
| Growth regulated oncogene-α (CXCL1) | GRO-α | 12 | 22.53(20.83-29.89) | GCST004457 |
| Granulocyte colony-stimulating factor | G-CSF | 9 | 22.54(20.94-25.07) | GCST004458 |
| Basic fibroblast growth factor | b-FGF | 7 | 22.04(20.79-25.40) | GCST004459 |
| Eotaxin (CCL11) | Eotaxin | 18 | 29.38(21.03-80.67) | GCST004460 |

Abbreviations: No., number; SNP, single nucleotide polymorphism.

**Table S3.** Effect estimates of the associations between circulating levels of 41 cytokines and telomere length in MR analyses.

| Methods | Number of SNPs | OR | 95% CI | *P-*value | *P-value |
| --- | --- | --- | --- | --- | --- |
| **β-NGF** |  |  |  |  |  |
| MR-Egger | 4 | 1.21 | 1.02-1.44 | 0.167 | *0.184 |
| Weighted median | 4 | 1.00 | 0.98-1.03 | 0.740 |  |
| Inverse-variance weighted | 4 | 1.01 | 0.97-1.06 | 0.534 |  |
| Simple mode | 4 | 1.00 | 0.97-1.02 | 0.847 |  |
| Weighted mode | 4 | 1.00 | 0.98-1.02 | 0.827 |  |
| **CTACK** |  |  |  |  |  |
| MR-Egger | 12 | 0.99 | 0.97-1.00 | 0.100 | *0.389 |
| Weighted median | 12 | 0.99 | 0.98-1.00 | 0.253 |  |
| Inverse-variance weighted | 12 | 0.99 | 0.98-1.00 | 0.069 |  |
| Simple mode | 12 | 0.99 | 0.98-1.01 | 0.426 |  |
| Weighted mode | 12 | 0.99 | 0.98-1.01 | 0.380 |  |
| **Eotaxin** |  |  |  |  |  |
| MR-Egger | 18 | 0.99 | 0.95-1.04 | 0.802 | *0.897 |
| Weighted median | 18 | 1.00 | 0.99-1.02 | 0.679 |  |
| Inverse-variance weighted | 18 | 1.00 | 0.98-1.01 | 0.705 |  |
| Simple mode | 18 | 1.01 | 0.97-1.04 | 0.761 |  |
| Weighted mode | 18 | 1.00 | 0.97-1.04 | 0.791 |  |
| **FGF-basic** |  |  |  |  |  |
| MR-Egger | 7 | 1.01 | 0.96-1.07 | 0.597 | *0.434 |
| Weighted median | 7 | 1.01 | 0.98-1.04 | 0.484 |  |
| Inverse-variance weighted | 7 | 0.99 | 0.97-1.02 | 0.615 |  |
| Simple mode | 7 | 1.00 | 0.96-1.03 | 0.906 |  |
| Weighted mode | 7 | 1.00 | 0.97-1.04 | 0.777 |  |
| **G-CSF** |  |  |  |  |  |
| MR-Egger | 9 | 1.01 | 0.99-1.03 | 0.379 | *0.496 |
| Weighted median | 9 | 1.01 | 1.00-1.03 | 0.107 |  |
| Inverse-variance weighted | 9 | 1.00 | 0.99-1.02 | 0.542 |  |
| Simple mode | 9 | 1.01 | 0.99-1.04 | 0.413 |  |
| Weighted mode | 9 | 1.01 | 0.99-1.03 | 0.205 |  |
| **GRO-a** |  |  |  |  |  |
| MR-Egger | 12 | 1.00 | 0.99-1.01 | 0.848 | *0.590 |
| Weighted median | 12 | 1.00 | 0.99-1.01 | 0.545 |  |
| Inverse-variance weighted | 12 | 1.00 | 0.99-1.01 | 0.697 |  |
| Simple mode | 12 | 0.99 | 0.98-1.01 | 0.310 |  |
| Weighted mode | 12 | 0.99 | 0.98-1.01 | 0.409 |  |
| **HGF** |  |  |  |  |  |
| MR-Egger | 9 | 1.03 | 1.00-1.06 | 0.101 | *0.025 |
| Weighted median | 9 | 1.00 | 0.98-1.02 | 0.894 |  |
| Inverse-variance weighted | 9 | 0.99 | 0.97-1.01 | 0.309 |  |
| Simple mode | 9 | 0.96 | 0.93-1.00 | 0.105 |  |
| Weighted mode | 9 | 1.01 | 0.98-1.03 | 0.689 |  |
| **IFN-γ** |  |  |  |  |  |
| MR-Egger | 12 | 0.98 | 0.95-1.00 | 0.120 | *0.176 |
| Weighted median | 12 | 0.99 | 0.97-1.01 | 0.360 |  |
| Inverse-variance weighted | 12 | 0.99 | 0.98-1.01 | 0.401 |  |
| Simple mode | 12 | 0.99 | 0.95-1.03 | 0.533 |  |
| Weighted mode | 12 | 0.98 | 0.95-1.01 | 0.307 |  |
| **IL-1rα** |  |  |  |  |  |
| MR-Egger | 10 | 1.00 | 0.96-1.04 | 0.982 | *0.810 |
| Weighted median | 10 | 1.01 | 0.99-1.02 | 0.371 |  |
| Inverse-variance weighted | 10 | 1.00 | 0.99-1.02 | 0.549 |  |
| Simple mode | 10 | 1.01 | 0.99-1.03 | 0.384 |  |
| Weighted mode | 10 | 1.01 | 0.99-1.03 | 0.479 |  |
| **IL-1β** |  |  |  |  |  |
| MR-Egger | 4 | 1.01 | 0.96-1.07 | 0.667 | *0.793 |
| Weighted median | 4 | 1.01 | 0.99-1.03 | 0.214 |  |
| Inverse-variance weighted | 4 | 1.01 | 0.99-1.03 | 0.565 |  |
| Simple mode | 4 | 1.01 | 0.99-1.04 | 0.382 |  |
| Weighted mode | 4 | 1.02 | 0.99-1.04 | 0.299 |  |
| **IL-2** |  |  |  |  |  |
| MR-Egger | 8 | 1.00 | 0.97-1.02 | 0.867 | *0.178 |
| Weighted median | 8 | 1.01 | 0.99-1.02 | 0.220 |  |
| Inverse-variance weighted | 8 | 1.01 | 1.00-1.03 | 0.061 |  |
| Simple mode | 8 | 1.01 | 0.99-1.04 | 0.428 |  |
| Weighted mode | 8 | 1.00 | 0.99-1.02 | 0.645 |  |
| **IL-2ra** |  |  |  |  |  |
| MR-Egger | 8 | 0.99 | 0.98-1.00 | 0.096 | *0.113 |
| Weighted median | 8 | 0.99 | 0.97-1.00 | 0.035 |  |
| Inverse-variance weighted | 8 | 0.98 | 0.96-1.00 | 0.045 |  |
| Simple mode | 8 | 0.98 | 0.96-1.00 | 0.152 |  |
| Weighted mode | 8 | 0.99 | 0.97-1.00 | 0.089 |  |
| **IL-4** |  |  |  |  |  |
| MR-Egger | 14 | 1.01 | 0.99-1.03 | 0.435 | *0.495 |
| Weighted median | 14 | 1.00 | 0.99-1.02 | 0.585 |  |
| Inverse-variance weighted | 14 | 1.00 | 0.99-1.01 | 0.681 |  |
| Simple mode | 14 | 0.99 | 0.96-1.01 | 0.320 |  |
| Weighted mode | 14 | 1.02 | 0.99-1.04 | 0.170 |  |
| **IL-5** |  |  |  |  |  |
| MR-Egger | 8 | 0.98 | 0.96-1.01 | 0.242 | *0.232 |
| Weighted median | 8 | 0.99 | 0.97-1.01 | 0.281 |  |
| Inverse-variance weighted | 8 | 1.00 | 0.99-1.01 | 0.824 |  |
| Simple mode | 8 | 0.99 | 0.96-1.01 | 0.354 |  |
| Weighted mode | 8 | 0.99 | 0.96-1.01 | 0.314 |  |
| **IL-6** |  |  |  |  |  |
| MR-Egger | 11 | 1.01 | 0.98-1.04 | 0.503 | *0.174 |
| Weighted median | 11 | 0.99 | 0.97-1.01 | 0.378 |  |
| Inverse-variance weighted | 11 | 0.99 | 0.97-1.01 | 0.305 |  |
| Simple mode | 11 | 0.99 | 0.96-1.02 | 0.368 |  |
| Weighted mode | 11 | 0.99 | 0.96-1.01 | 0.342 |  |
| **IL-7** |  |  |  |  |  |
| MR-Egger | 14 | 1.00 | 0.98-1.02 | 0.783 | *0.139 |
| Weighted median | 14 | 1.01 | 1.00-1.02 | 0.227 |  |
| Inverse-variance weighted | 14 | 1.01 | 1.00-1.02 | 0.032 |  |
| Simple mode | 14 | 1.01 | 0.99-1.02 | 0.385 |  |
| Weighted mode | 14 | 1.01 | 0.99-1.02 | 0.321 |  |
| **IL-8** |  |  |  |  |  |
| MR-Egger | 8 | 1.00 | 0.98-1.01 | 0.791 | *0.429 |
| Weighted median | 8 | 1.00 | 0.99-1.01 | 0.647 |  |
| Inverse-variance weighted | 8 | 1.00 | 0.99-1.01 | 0.561 |  |
| Simple mode | 8 | 1.00 | 0.99-1.02 | 0.680 |  |
| Weighted mode | 8 | 1.00 | 0.99-1.02 | 0.695 |  |
| **IL-9** |  |  |  |  |  |
| MR-Egger | 6 | 1.00 | 0.97-1.03 | 0.917 | *0.920 |
| Weighted median | 6 | 1.00 | 0.99-1.02 | 0.865 |  |
| Inverse-variance weighted | 6 | 1.00 | 0.99-1.01 | 0.973 |  |
| Simple mode | 6 | 1.01 | 0.98-1.03 | 0.608 |  |
| Weighted mode | 6 | 1.01 | 0.98-1.03 | 0.607 |  |
| **IL-10** |  |  |  |  |  |
| MR-Egger | 16 | 0.99 | 0.97-1.02 | 0.478 | *0.279 |
| Weighted median | 16 | 1.00 | 0.99-1.02 | 0.862 |  |
| Inverse-variance weighted | 16 | 1.00 | 0.99-1.02 | 0.573 |  |
| Simple mode | 16 | 1.00 | 0.97-1.03 | 0.868 |  |
| Weighted mode | 16 | 1.00 | 0.97-1.02 | 0.685 |  |
| **IL-12p70** |  |  |  |  |  |
| MR-Egger | 15 | 0.96 | 0.91-1.01 | 0.160 | *0.085 |
| Weighted median | 15 | 1.01 | 0.99-1.03 | 0.350 |  |
| Inverse-variance weighted | 15 | 1.01 | 0.99-1.02 | 0.282 |  |
| Simple mode | 15 | 1.02 | 0.99-1.05 | 0.280 |  |
| Weighted mode | 15 | 1.01 | 0.98-1.03 | 0.567 |  |
| **IL-13** |  |  |  |  |  |
| MR-Egger | 12 | 0.99 | 0.98-1.01 | 0.469 | *0.655 |
| Weighted median | 12 | 1.00 | 0.99-1.01 | 0.77 3 |  |
| Inverse-variance weighted | 12 | 1.00 | 0.99-1.01 | 0.529 |  |
| Simple mode | 12 | 0.98 | 0.96-1.00 | 0.091 |  |
| Weighted mode | 12 | 1.00 | 0.99-1.02 | 0.667 |  |
| **IL-16** |  |  |  |  |  |
| MR-Egger | 10 | 0.99 | 0.98-1.01 | 0.439 | *0.823 |
| Weighted median | 10 | 1.00 | 0.98-1.01 | 0.381 |  |
| Inverse-variance weighted | 10 | 1.00 | 0.99-1.00 | 0.294 |  |
| Simple mode | 10 | 0.99 | 0.97-1.01 | 0.329 |  |
| Weighted mode | 10 | 1.00 | 0.98-1.01 | 0.540 |  |
| **IL-17** |  |  |  |  |  |
| MR-Egger | 8 | 1.01 | 0.98-1.04 | 0.447 | *0.551 |
| Weighted median | 8 | 1.00 | 0.98-1.02 | 0.797 |  |
| Inverse-variance weighted | 8 | 1.00 | 0.99-1.02 | 0.597 |  |
| Simple mode | 8 | 0.99 | 0.96-1.02 | 0.489 |  |
| Weighted mode | 8 | 0.99 | 0.97-1.02 | 0.639 |  |
| **IL-18** |  |  |  |  |  |
| MR-Egger | 14 | 1.00 | 0.99-1.01 | 0.989 | *0.752 |
| Weighted median | 14 | 1.00 | 0.99-1.01 | 0.814 |  |
| Inverse-variance weighted | 14 | 1.00 | 0.99-1.01 | 0.668 |  |
| Simple mode | 14 | 1.00 | 0.99-1.02 | 0.617 |  |
| Weighted mode | 14 | 1.00 | 0.99-1.02 | 0.717 |  |
| **IP-10** |  |  |  |  |  |
| MR-Egger | 11 | 0.99 | 0.97-1.02 | 0.478 | *0.279 |
| Weighted median | 11 | 1.00 | 0.99-1.02 | 0.862 |  |
| Inverse-variance weighted | 11 | 1.00 | 0.99-1.02 | 0.573 |  |
| Simple mode | 11 | 1.00 | 0.97-1.03 | 0.868 |  |
| Weighted mode | 11 | 1.00 | 0.97-1.02 | 0.685 |  |
| **MCP-1** |  |  |  |  |  |
| MR-Egger | 17 | 1.00 | 0.97-1.03 | 0.983 | *0.839 |
| Weighted median | 17 | 1.00 | 0.99-1.02 | 0.609 |  |
| Inverse-variance weighted | 17 | 1.00 | 0.98-1.01 | 0.712 |  |
| Simple mode | 17 | 1.01 | 0.97-1.04 | 0.735 |  |
| Weighted mode | 17 | 1.01 | 0.98-1.04 | 0.490 |  |
| **MCP-3** |  |  |  |  |  |
| MR-Egger | 6 | 1.01 | 0.99-1.03 | 0.247 | *0.226 |
| Weighted median | 6 | 1.00 | 0.99-1.01 | 0.904 |  |
| Inverse-variance weighted | 6 | 1.00 | 0.99-1.01 | 0.915 |  |
| Simple mode | 6 | 0.99 | 0.97-1.01 | 0.414 |  |
| Weighted mode | 6 | 0.99 | 0.97-1.01 | 0.420 |  |
| **M-CSF** |  |  |  |  |  |
| MR-Egger | 12 | 1.00 | 0.98-1.02 | 0.973 | *0.613 |
| Weighted median | 12 | 1.00 | 0.99-1.01 | 0.889 |  |
| Inverse-variance weighted | 12 | 1.00 | 1.00-1.01 | 0.365 |  |
| Simple mode | 12 | 1.00 | 0.99-1.01 | 0.976 |  |
| Weighted mode | 12 | 1.00 | 0.99-1.01 | 0.922 |  |
| **MIF** |  |  |  |  |  |
| MR-Egger | 10 | 0.99 | 0.97-1.01 | 0.184 | *0.201 |
| Weighted median | 10 | 0.99 | 0.98-1.00 | 0.144 |  |
| Inverse-variance weighted | 10 | 1.00 | 0.99-1.01 | 0.607 |  |
| Simple mode | 10 | 0.99 | 0.97-1.01 | 0.352 |  |
| Weighted mode | 10 | 0.99 | 0.97-1.01 | 0.271 |  |
| **MIG** |  |  |  |  |  |
| MR-Egger | 12 | 1.00 | 0.98-1.03 | 0.789 | *0.951 |
| Weighted median | 12 | 1.00 | 0.99-1.02 | 0.855 |  |
| Inverse-variance weighted | 12 | 1.00 | 0.99-1.02 | 0.652 |  |
| Simple mode | 12 | 1.00 | 0.98-1.02 | 0.956 |  |
| Weighted mode | 12 | 1.00 | 0.98-1.02 | 0.818 |  |
| **MIP-1α** |  |  |  |  |  |
| MR-Egger | 4 | 1.03 | 0.95-1.12 | 0.553 | *0.584 |
| Weighted median | 4 | 1.01 | 0.99-1.03 | 0.427 |  |
| Inverse-variance weighted | 4 | 1.00 | 0.98-1.03 | 0.743 |  |
| Simple mode | 4 | 1.02 | 0.98-1.06 | 0.429 |  |
| Weighted mode | 4 | 1.02 | 0.98-1.06 | 0.405 |  |
| **MIP-1β** |  |  |  |  |  |
| MR-Egger | 23 | 1.01 | 0.98-1.03 | 0.545 | *0.805 |
| Weighted median | 23 | 1.00 | 0.99-1.02 | 0.742 |  |
| Inverse-variance weighted | 23 | 1.00 | 1.00-1.01 | 0.312 |  |
| Simple mode | 23 | 1.00 | 0.97-1.02 | 0.829 |  |
| Weighted mode | 23 | 1.00 | 0.98-1.02 | 0.915 |  |
| **PDGF-bb** |  |  |  |  |  |
| MR-Egger | 14 | 1.00 | 0.97-1.03 | 0.919 | *0.707 |
| Weighted median | 14 | 1.01 | 0.99-1.03 | 0.536 |  |
| Inverse-variance weighted | 14 | 1.01 | 0.99-1.02 | 0.320 |  |
| Simple mode | 14 | 0.99 | 0.96-1.02 | 0.526 |  |
| Weighted mode | 14 | 1.00 | 0.97-1.03 | 0.896 |  |
| **RANTES** |  |  |  |  |  |
| MR-Egger | 9 | 1.00 | 0.98-1.03 | 0.789 | *0.926 |
| Weighted median | 9 | 1.00 | 0.99-1.02 | 0.861 |  |
| Inverse-variance weighted | 9 | 1.00 | 0.99-1.01 | 0.657 |  |
| Simple mode | 9 | 1.00 | 0.98-1.02 | 0.884 |  |
| Weighted mode | 9 | 1.00 | 0.98-1.02 | 0.883 |  |
| **SCF** |  |  |  |  |  |
| MR-Egger | 10 | 1.04 | 1.00-1.08 | 0.074 | *0.161 |
| Weighted median | 10 | 1.01 | 0.99-1.03 | 0.156 |  |
| Inverse-variance weighted | 10 | 1.01 | 0.99-1.03 | 0.189 |  |
| Simple mode | 10 | 1.02 | 0.99-1.05 | 0.317 |  |
| Weighted mode | 10 | 1.01 | 0.99-1.04 | 0.313 |  |
| **SCGF-β** |  |  |  |  |  |
| MR-Egger | 21 | 1.01 | 1.00-1.03 | 0.145 | *0.287 |
| Weighted median | 21 | 1.01 | 1.00-1.02 | 0.153 |  |
| Inverse-variance weighted | 21 | 1.00 | 1.00-1.01 | 0.243 |  |
| Simple mode | 21 | 1.01 | 0.99-1.03 | 0.195 |  |
| Weighted mode | 21 | 1.01 | 0.99-1.02 | 0.287 |  |
| **SDF-1α** |  |  |  |  |  |
| MR-Egger | 10 | 1.01 | 0.98-1.03 | 0.672 | *0.954 |
| Weighted median | 10 | 1.00 | 0.98-1.02 | 0.919 |  |
| Inverse-variance weighted | 10 | 1.01 | 0.99-1.02 | 0.408 |  |
| Simple mode | 10 | 1.00 | 0.98-1.03 | 0.762 |  |
| Weighted mode | 10 | 1.00 | 0.98-1.02 | 0.912 |  |
| **TNF-α** |  |  |  |  |  |
| MR-Egger | 5 | 0.99 | 0.97-1.01 | 0.447 | *0.183 |
| Weighted median | 5 | 1.00 | 0.99-1.02 | 0.655 |  |
| Inverse-variance weighted | 5 | 1.01 | 0.99-1.02 | 0.443 |  |
| Simple mode | 5 | 1.00 | 0.98-1.03 | 0.708 |  |
| Weighted mode | 5 | 1.00 | 0.98-1.02 | 0.817 |  |
| **TNF-β** |  |  |  |  |  |
| MR-Egger | 5 | 1.00 | 0.98-1.02 | 0.929 | *0.432 |
| Weighted median | 5 | 1.00 | 0.99-1.01 | 0.504 |  |
| Inverse-variance weighted | 5 | 1.01 | 1.00-1.01 | 0.192 |  |
| Simple mode | 5 | 1.00 | 0.99-1.02 | 0.680 |  |
| Weighted mode | 5 | 1.00 | 0.99-1.02 | 0.664 |  |
| **TRAIL** |  |  |  |  |  |
| MR-Egger | 15 | 1.00 | 0.99-1.01 | 0.800 | *0.417 |
| Weighted median | 15 | 1.00 | 0.99-1.01 | 0.557 |  |
| Inverse-variance weighted | 15 | 1.00 | 0.99-1.01 | 0.647 |  |
| Simple mode | 15 | 1.01 | 0.99-1.03 | 0.570 |  |
| Weighted mode | 15 | 1.00 | 0.99-1.01 | 0.973 |  |
| **VEGF** |  |  |  |  |  |
| MR-Egger | 17 | 1.00 | 0.99-1.02 | 0.898 | *0.248 |
| Weighted median | 17 | 1.00 | 0.98-1.01 | 0.668 |  |
| Inverse-variance weighted | 17 | 0.99 | 0.98-1.00 | 0.157 |  |
| Simple mode | 17 | 1.00 | 0.98-1.02 | 0.845 |  |
| Weighted mode | 17 | 1.00 | 0.98-1.01 | 0.732 |  |

Abbreviations: β-NGF, beta nerve growth factor; CTACK, cutaneous T-cell attracting (CCL27); FGF-basic, basic fibroblast growth factor; G-CSF, granulocyte colony-stimulating factor; GRO-a, growth regulated oncogene-α (CXCL1); HGF, hepatocyte growth factor; IFN-γ, interferon-gamma; IL-1rα, interleukin-1 receptor antagonist; IL-1β, interleukin-1-beta; IL-2, interleukin-2; IL-2rα, interleukin-2 receptor, alpha subunit; IL-4, interleukin-4; IL-5, interleukin-5; IL-6, interleukin-6; IL-7, interleukin-7; IL-8, interleukin-8; IL-9, interleukin-9; IL-10, interleukin-10; IL-12p70, interleukin-12p70; IL-13, interleukin-13; IL-16, interleukin-16; IL-17, interleukin-17; IL-18, interleukin-18; IP-10, interferon gamma-induced protein 10 (CXCL10); MCP-1, monocyte chemotactic protein-1; MCP-3, monocyte specific chemokine 3 (CCL7); M-CSF, macrophage colony-stimulating factor; MIF, macrophage migration inhibitory factor; MIG, monokine induced by interferon-gamma; MIP-1α, macrophage inflammatory protein-1α (CCL3); MIP-1b, macrophage inflammatory protein-1β; MR; PDGF-bb, platelet derived growth factor BB; RANTES, regulated on activation normal T Cell expressed and secreted (CCL5); SCF, stem cell factor; SCGF-β, stem cell growth factor beta; SDF-1α, stromal cell-derived factor-1 alpha; SNP, single nucleotide polymorphism; TNF-α, tumor necrosis factor-alpha; TNF-β, tumor necrosis factor-beta; TRAIL, TNF-related apoptosis inducing ligand; VEGF, vascular endothelial growth factor; CI, confidence interval; OR, odds ratio; SNP, single nucleotide polymorphism. **P*-value of the intercept from MR-Egger regression analysis.

**Table S4.** Characteristics of instrumental variables used for circulating levels of IL-7 and IL-2Rα in this study.

| Cytokines/ Growth factors | SNP | Chr | Position | Effect allele | Beta | SE | *P*-value |
| --- | --- | --- | --- | --- | --- | --- | --- |
| IL-7 | rs115215018 | 4 | 181079135 | T | 0.5985 | 0.1308 | 4.76×10^-06^ |
| IL-7 | rs117509142 | 8 | 87134083 | T | -0.3213 | 0.0684 | 2.60×10^-06^ |
| IL-7 | rs11757972 | 6 | 43858592 | T | 0.121 | 0.0257 | 2.53×10^-06^ |
| IL-7 | rs1374279 | 2 | 168652203 | A | 0.1625 | 0.0347 | 2.79×10^-06^ |
| IL-7 | rs142397827 | 5 | 17484169 | A | 0.4592 | 0.0994 | 3.82×10^-06^ |
| IL-7 | rs17091524 | 14 | 56948759 | T | 0.5092 | 0.1015 | 5.24×10^-07^ |
| IL-7 | rs2006957 | 6 | 43913243 | T | 0.2557 | 0.0262 | 1.43×10^-22^ |
| IL-7 | rs218238 | 4 | 55395024 | A | 0.1319 | 0.0284 | 3.28×10^-06^ |
| IL-7 | rs28793375 | 8 | 41415618 | T | 0.1644 | 0.036 | 4.87×10^-06^ |
| IL-7 | rs62006410 | 14 | 103007935 | T | -0.1492 | 0.0302 | 7.59×10^-07^ |
| IL-7 | rs7155170 | 14 | 30338797 | A | -0.1236 | 0.027 | 4.79×10^-06^ |
| IL-7 | rs77318030 | 19 | 55055897 | T | -0.2966 | 0.0631 | 2.64×10^-06^ |
| IL-7 | rs77981494 | 16 | 17544866 | T | -0.5201 | 0.1055 | 8.23×10^-07^ |
| IL-7 | rs78346957 | 10 | 126903513 | A | 0.4632 | 0.1008 | 4.30×10^-06^ |
| IL-2Rα | rs11241559 | 5 | 119976700 | T | -0.124 | 0.0264 | 2.75×10^-06^ |
| IL-2Rα | rs117244812 | 17 | 6443310 | A | -0.7187 | 0.1493 | 1.47×10^-06^ |
| IL-2Rα | rs12789243 | 11 | 123374923 | T | 0.1263 | 0.0276 | 4.61×10^-06^ |
| IL-2Rα | rs17147986 | 10 | 6038478 | A | -0.2976 | 0.0337 | 1.07×10^-18^ |
| IL-2Rα | rs17624670 | 8 | 32126374 | A | -0.125 | 0.0273 | 4.64×10^-06^ |
| IL-2Rα | rs34037190 | 10 | 5919360 | A | 0.4784 | 0.0935 | 3.13×10^-07^ |
| IL-2Rα | rs56213152 | 7 | 100664615 | T | 0.1269 | 0.0271 | 2.94×10^-06^ |
| IL-2Rα | rs79100208 | 3 | 59351505 | C | 0.8345 | 0.1758 | 2.05×10^-06^ |

Abbreviations: Chr, chromosome; IL-7, interleukin-7; IL-2Rα, Interleukin-2 receptor, alpha subunit; SE, standard error; SNP, single nucleotide polymorphism.

**Table S5.** The global test and causal estimate results of MR pleiotropy residual sum and outlier (MR-PRESSO).

| Cytokines/ Growth factors | Causal estimate *P*-value | Global test *P*-value | Pleiotropic SNP |  |
| --- | --- | --- | --- | --- |
| CTACK | 0.088 | 0.507 | no outlier |  |
| β-NGF | 0.135 | 0.569 | no outlier |  |
| VEGF | 0.232 | 0.963 | no outlier |  |
| MIF | 0.369 | 0.843 | no outlier |  |
| TRAIL | 0.280 | 0.735 | no outlier |  |
| TNF-β | 0.021 | 0.836 | no outlier |  |
| TNF-α | 0.538 | 0.632 | no outlier |  |
| SDF-1α | 0.368 | 0.394 | no outlier |  |
| SCGF-β | 0.278 | 0.434 | no outlier |  |
| SCF | 0.222 | 0.077 | no outlier |  |
| IL-16 | 0.511 | 0.345 | no outlier |  |
| RANTES | 0.617 | 0.318 | no outlier |  |
| PDGF-bb | 0.032 | 0.723 | no outlier |  |
| MIP-1β | 0.018 | 0.228 | no outlier |  |
| MIP-1α | 0.505 | 0.476 | no outlier |  |
| MIG | 0.511 | 0.065 | no outlier |  |
| MCSF | 0.047 | 0.676 | no outlier |  |
| MCP-3 | 0.345 | 0.323 | no outlier |  |
| MCP-1 | 0.585 | 0.119 | no outlier |  |
| IL-12p70 | 0.939 | 0.321 | no outlier |  |
| IP-10 | 0.185 | 0.484 | no outlier |  |
| IL-18 | 0.766 | 0.720 | no outlier |  |
| IL-17 | 0.452 | 0.406 | no outlier |  |
| IL-13 | 0.494 | 0.798 | no outlier |  |
| IL-10 | 0.930 | 0.237 | no outlier |  |
| IL-8 | 0.122 | 0.925 | no outlier |  |
| IL-6 | 0.354 | 0.590 | no outlier |  |
| IL-1rα | 0.635 | 0.860 | no outlier |  |
| IL-1β | 0.00024 | 0.966 | no outlier |  |
| HGF | 0.651 | 0.307 | no outlier |  |
| IL-9 | 0.423 | 0.735 | no outlier |  |
| IL-7 | 0.388 | 0.152 | no outlier |  |
| IL-5 | 0.977 | 0.222 | no outlier |  |
| IL-4 | 0.509 | 0.662 | no outlier |  |
| IL-2Rα | 0.250 | 0.496 | no outlier |  |
| IL-2 | 0.398 | 0.147 | no outlier |  |
| IFN-γ | 0.796 | 0.058 | no outlier |  |
| GRO-α | 0.554 | 0.917 | no outlier |  |
| G-CSF | 0.375 | 0.758 | no outlier |  |
| b-FGF | 0.343 | 0.264 | no outlier |  |
| Eotaxin | 0.347 | 0.069 | no outlier |  |

SNP, single nucleotide polymorphism.

**Table S6.** Effect estimates of the associations of telomere length with circulating levels of IL-7 and IL-2rα.

| Methods | Number of SNPs | OR | 95% CI | *P-*value（*P-value） |
| --- | --- | --- | --- | --- |
| IL-7 |  |  |  |  |
| MR-Egger | 117 | 0.95 | 0.68-1.33 | 0.770（*0.974） |
| Weighted median | 117 | 0.98 | 0.71-1.35 | 0.889 |
| Inverse-variance weighted | 117 | 0.95 | 0.78-1.16 | 0.647 |
| Simple mode | 117 | 0.78 | 0.37-1.61 | 0.498 |
| Weighted mode | 117 | 1.09 | 1.72-1.64 | 0.690 |
| IL-2rα |  |  |  |  |
| MR-Egger | 117 | 0.93 | 0.67-1.30 | 0.680（*0.509） |
| Weighted median | 117 | 0.85 | 0.63-1.15 | 0.299 |
| Inverse-variance weighted | 117 | 0.85 | 0.70-1.03 | 0.104 |
| Simple mode | 117 | 0.76 | 0.40-1.43 | 0.398 |
| Weighted mode | 117 | 0.85 | 0.61-1.19 | 0.343 |

Abbreviations: CI, confidence interval; IL-7, interleukin-7; IL-2rα, Interleukin-2 receptor, alpha subunit; OR, odds ratio; SNP, single nucleotide polymorphism. **P*-value of the intercept from MR-Egger regression analysis.
